# Evolution of Support Vector Machine and Regression Modeling in Chemoinformatics and Drug Discovery

**DOI:** 10.1007/s10822-022-00442-9

**Published:** 2022-03-19

**Authors:** Raquel Rodríguez-Pérez, Jürgen Bajorath

**Affiliations:** 1 Department of Life Science Informatics, B-IT, LIMES Program Unit Chemical Biology and Medicinal Chemistry, Rheinische Friedrich-Wilhelms-Universität, Friedrich-Hirzebruch-Allee 6, D-53115 Bonn, Germany; 2 Novartis Institutes for Biomedical Research, Novartis Campus, CH-4002 Basel, Switzerland

**Keywords:** Support vector machines, Machine learning, Compound classification, Property prediction, Regression

## Abstract

The support vector machine (SVM) algorithm is one of the most widely used machine learning (ML) methods for predicting active compounds and molecular properties. In chemoinformatics and drug discovery, SVM has been a state-of-the-art ML approach for more than a decade. A unique attribute of SVM is that it operates in feature spaces of increasing dimensionality. Hence, SVM conceptually departs from the paradigm of low dimensionality that applies to many other methods for chemical space navigation. The SVM approach is applicable to compound classification, and ranking, multi-class predictions, and –in algorithmically modified form– regression modeling. In the emerging era of deep learning (DL), SVM retains its relevance as one of the premier ML methods in chemoinformatics, for reasons discussed herein. We describe the SVM methodology including strengths and weaknesses and discuss selected applications that have contributed to the evolution of SVM as a premier approach for compound classification, property predictions, and virtual compound screening.

## Introduction

The support vector machine (SVM) concept was introduced by Vapnik in 1979 [1, 2]. The approach was originally designed for binary object classification and then adapted for the prediction of numerical values (termed support vector regression, SVR). The algorithm projects training data into a pre-defined feature space to derive a model for qualitative or quantitative predictions by searching for a hyperplane that best separates positive and negative training instances (SVM) or by fitting a regression function (SVR). A unique feature of SVM setting it apart from other machine learning (ML) methods is that it operates in features spaces of increasing dimensionality to search for hyperplanes that linearly separate positive and negative training data. Accordingly, if linear separation is not possible in a given feature space, the data are mapped into a higher-dimensional space where linear separation might become feasible.

The soft margin classifier variant of SVM that is widely used at present and SVR received increasing attention during the 1990s [3–5] and were beginning to be applied in chemoinformatics in the early 2000s [6–8]. Subsequently, SVM/SVR became one of the most popular ML approaches in chemoinformatics and drug discovery together with decision tree-based methods such as random forests (RF) [9] and probabilistic approaches such as Bayesian modeling [10]. These methods essentially replaced (shallow) neural networks (NNs) for applications such as compound activity/property predictions [11]. This was the case because NNs were prone to overfitting using available training sets of limited size and had less generalization potential than SVM or RF. These algorithms continue to be mainstays in chemoinformatics and drug discovery while deep learning (DL) using deep NN (DNN) architectures has been increasingly applied in recent years [12, 13]. To us, SVM is of particular interest, given its conceptual elegance, methodological uniqueness, versatility, and consistently high performance in many chemoinformatics applications, as discussed in the following.

## Methodological foundations

We begin with a few general definitions that should be helpful to follow the theory discussion.

A *hyperplane* is defined as a subspace with one dimension less than the N-dimensional feature space in which it is formed. In SVM modeling, the hyperplane represents a classification boundary. The *margin* of the hyperplane is the distance between two object classes in feature space separated by the hyperplane for SVM classification. *Support vectors (SVs)* represent data samples of one class that are closest to the other class and thus used to define the margin of the hyperplane. *Kernel function* is a similarity function that takes as input vectors in original feature space and calculates a modified inner product in a higher-dimensional space. The *kernel trick* refers to a strategy for generating a non-linear SVM using a kernel function instead of computing an explicit mapping of data into a higher-dimensional space. The *ε-insensitive tube* in SVR is equivalent to the margin in SVM classification and indicates the deviations that are tolerated in the prediction of numerical values. Deviations larger than ε are penalized. Support vectors in SVR correspond to data points falling outside the ε-tube.

**Fig. 1:**
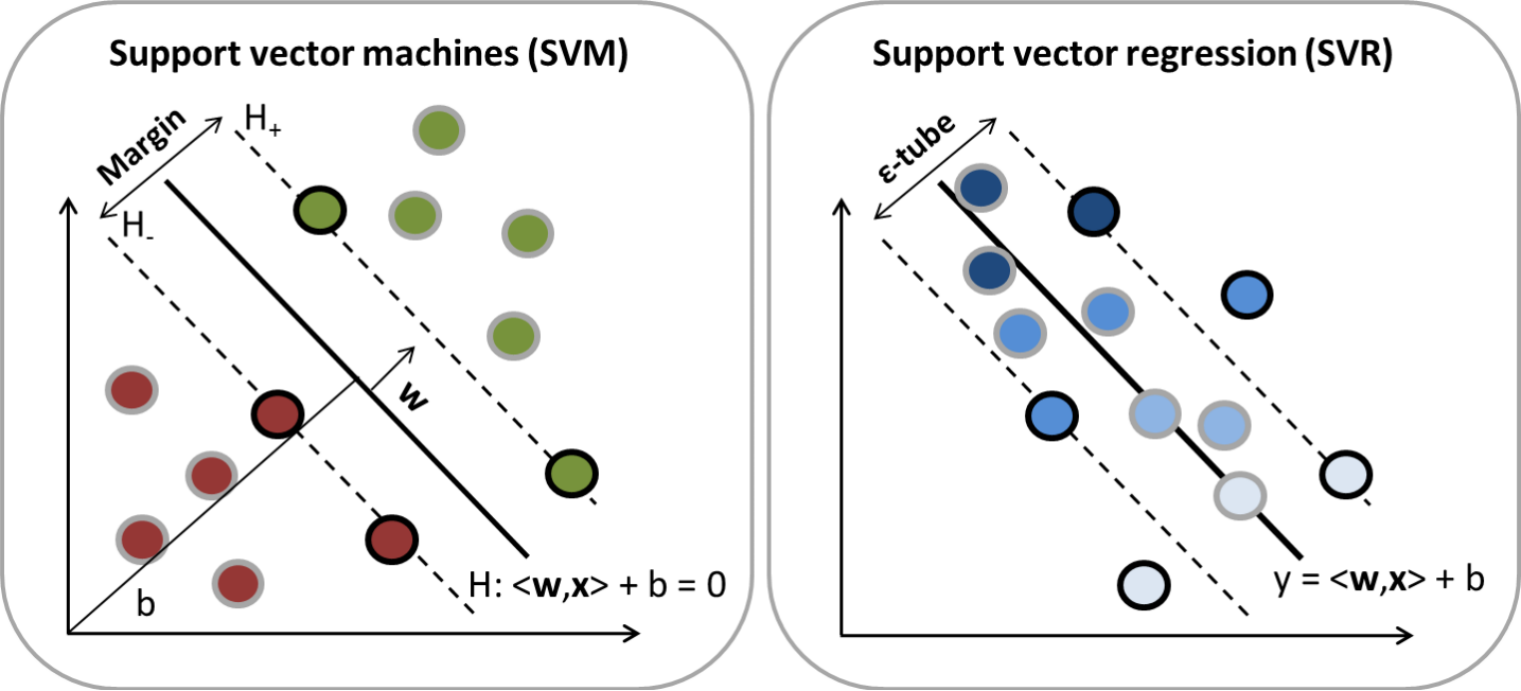
**SVM and SVR modeling.** In SVM (left), a hyperplane with maximal margin is constructed to separate two compound classes (colored green and red, respectively). In SVR (right), the difference between an observed and predicted numerical value is minimized. The gradient from dark to light blue indicates decreasing numerical values. Support vectors for SVM/SVR are indicated by black circles. In SVM, SVs are located on the margin, while they may be located outside of the ε-tube in SVR

SVM is a supervised ML algorithm that can be used for compound classification and ranking and SVR is an extension of SVM that is used for predicting numerical values. SVM and SVR learning is schematically compared in Fig. 1. In SVM, model building relies on the derivation of the SVs that are differently defined for classification and regression, as illustrated in Fig. 1. Both strategies balance the risk of model overfitting to training data, which generally hinders generalization of ML models.

SVM uses labeled training data to define a hyperplane as a classification border between two object classes. As any supervised learning model, an SVM model is derived from a data matrix containing the molecular features (descriptors) for training compounds and a vector with their activity class labels. Molecular features are organized in a data matrix $$X\in {\mathbb{R}}^{D}$$, in which rows correspond to different compounds and columns to different molecular descriptors. In addition, the class label of each compound $$\mathbf{x}\in X$$ is represented by a binary categorical vector which indicates the class label $$y\in \{-1,+1\}$$, i.e. inactivity (-1) or activity (+1). SVM projects these training data into a feature space $$\mathcal{X}$$ (defined by the molecular representation) and constructs a hyperplane $$H$$ that optimally separates the two classes under study, shown on the left in Fig. 1. A weight vector **w** and a bias *b* are used to define this hyperplane as $$H = \left\{ {{\bf{x}}| < {\bf{w}},{\bf{x}} > + b = 0} \right\}$$ where <∙,∙> is a scalar product [4]. Once $$H$$ is derived, test data can be projected into the input space and classified or ranked according to their position with respect to $$H$$. Hence, test compounds are predicted to be positive (active) or negative (inactive), depending on the side of the hyperplane onto which they fall. Alternatively, test instances can be ranked according to the signed (positive or negative) distance from the hyperplane by assigning a distance-based probability of activity to compounds. SVs are samples of one class that are closest to the other (indicated by black circles in Fig. 1) and represent the subset of training instances from which the hyperplane is derived. The distance between SVs from each class is known as margin ($${H}_{+}-{H}_{-})$$ and the SVM objective function aims to maximize the margin. However, a hyperplane that preferentially maximizes the margin is prone to overfitting, one of the major pitfalls in ML. Such models would only be suitable for predicting training data, but lack predictive potential for test data. To avoid overfitting in SVM modeling, non-negative slack variables are added to the optimization function to permit limited training errors (i.e., some training data points may fall onto the incorrect side of the hyperplane or within the margin). Relaxation of margin maximization is controlled by the hyper-parameter C (regularization term or cost factor), which introduces a trade-off between margin size and classification error. Smaller C values cause a larger margin, which results in a simpler model with worse training set predictions, whereas larger C values lead to a smaller margin and better predictive performance on training data. Note again that perfect training set predictions do not guarantee generalization ability of the model and likely cause model overfitting. Generally, the cost factor C accepts values ranging from 0.001 to 1000 and is optimized using cross-validation on the training data.

SVR enables the prediction of numerical property values. Regression models are built from training data $X\in {\mathbb{R}}^{D}$ and a numerical vector containing the property value $y\in \mathbb{R}$ of each training compound. SVR defines a regression function of the form $f\left( {\bf{x}} \right) = < {\bf{w}},{\bf{x}} > + b$ and attempts to map training data as closely as possible to the numerical label, illustrated in Fig. 1 (right). Limited derivations from precise values are tolerated by the ε-insensitive tube [5], whereas errors larger than ε are penalized. Hence, ε defines the tolerance limits for differences between predicted and real values of training instances. Analogously to SVM, non-negative slack variables are introduced to permit few training instances to fall outside the ε-tube, which represent SVs for deriving the model. Furthermore, the regularization term C is also introduced in SVR to balance complexity and accuracy of the model. For a large value of C, a complex model is obtained that avoids training errors with the risk of overfitting, whereas a small value of C leads to a model of low complexity with a tendency to insufficiently fit training data [14]. Hence, arriving at a well-balanced setting of C is critical for achieving a meaningful compromise between model accuracy and generalizability. The C value is typically selected on the basis of cross-validation results.

**Fig. 2:**
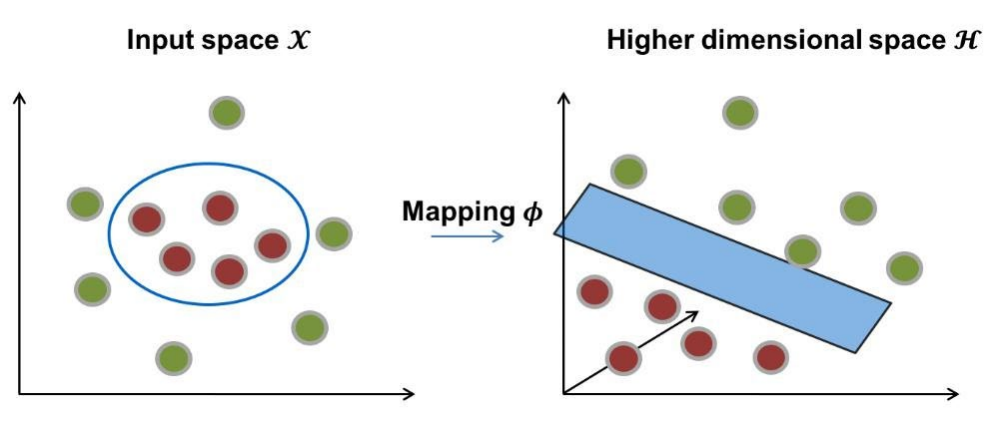
**Kernel trick.** If two classes of objects cannot be linearly separated in a given feature space *X*, a non-linear mapping *ɸ* is performed to project data points into a higher-dimensional space $\mathcal{H}$ in which a linear hyperplane separating positive and negative instances might be found. The kernel trick circumvents the explicit mapping through the use of kernel functions

The “kernel trick” plays a central role in SVM/SVR modeling. If data is non-linearly separable or a linear regression is not possible in a given input space $\mathcal{X}$, the kernel trick is applied to map the training data into a higher dimensional space $\mathcal{H}$ in which linear separation might be feasible [15]. Thus, a linear model is built in the new space $\mathcal{H}$, which corresponds to a non-linear model in $\mathcal{X}$, as shown in Fig. 2. The scalar product is transferred into a higher dimensional space by a nonlinear transformation $\varphi$, but the explicit mapping is not computed. Instead, the scalar product is replaced by a kernel function K(∙,∙). Among the most common kernel functions are the linear kernel (equivalent to the original scalar product), the Gaussian or Radial Basis Function (RBF) kernel (*Eq. 1*), or the polynomial kernel (*Eq. 2*). Here, $\gamma$ and *d* are hyper-parameters of the algorithm and *u, v* two vectors.

1 $${K_{RBF}}\left( {u,v} \right) = {\rm{exp}}( - \gamma {\left\| {u\left. { - v} \right\|} \right.^2})$$

2 $${K_{polynomial}}(u,v) = {( < u,v > + 1)^d}$$

Moreover, the Tanimoto kernel (*Eq. 3*), which is based on the Tanimoto coefficient for quantifying the similarity of vector representations, has become especially popular for applications in chemoinformatics [16].

3 $${K_{Tanimoto}}(u,v) = \frac{{ < u,v > }}{{ < u,u > + < v,v > - < u,v > }}$$

Importantly, the use of kernel functions enables mapping into higher dimensional feature spaces without the need to compute the explicit space transformation, which is a hallmark of SVM and SVR modeling.

## Selected applications

SVM became an ML method of choice in chemoinformatics because it typically achieved high accuracy in compound classification (class label prediction), which was of particular relevance for virtual compound screening (VS) [17]. Subsequently, SVR was established as a primary approach for non-linear QSAR and also applied for VS [17, 18]. In ML-based VS, classification or regression models are built to distinguish between known active and inactive (or randomly selected) compounds and used to screen databases [17–19]. For SVM, compound rankings can then be generated in the order of decreasing likelihood of activity based upon the signed distance of test compounds from the hyperplane (see above). In SVR, test compounds can be ranked by predicted potency values. Different studies have highlighted the potential of SVM and SVR to detect structurally novel active compounds distinct from those used for training [e.g. 7, 20–24]. For example, SVM was combined with an active learning strategy to identify thrombin inhibitors [7] and new inhibitors of histone deacetylase 1 (HDACI1) were predicted by SVR screening of ~ 9.5 million compounds and experimentally confirmed [24]. In chemoinformatics, VS represents a standard application and many retrospective or prospective VS applications using SVM/SVR (and other state-of-the-art ML approaches) have been reported over the years. Furthermore, SVM modeling was adapted for a variety of special applications, some of which are discussed below.

### Multi-target activities

**Fig. 3:**
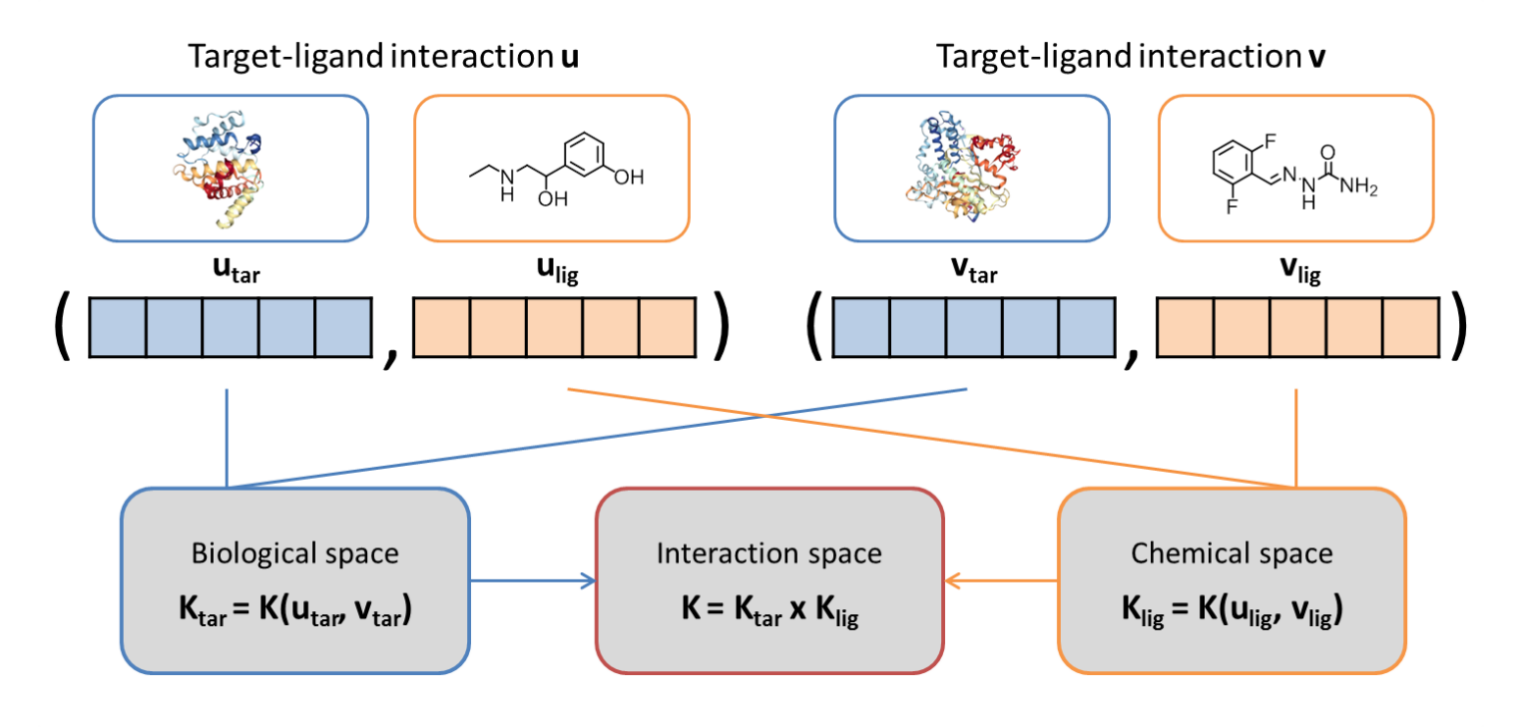
**Target-ligand kernel.** Ligand (orange) and target (blue) descriptors are concatenated to represent an interaction. Two kernel functions are used to separately calculate target and compound similarity. Then, the product kernel is calculated for target-ligand pairings yielding a combined similarity score

In addition to predicting target-specific compound activity, multi-target activities can also be predicted. There are several ways in which multi-class SVM modeling can be facilitated including the *one-vs-all* (i.e., one classifier per target), *one-vs-one* (one classifier per pair of targets, followed by selection of the target with strongest predictive support), or *classifier chain* strategy [24]. In a *classifier chain*, sequences of single-target classifiers are built by iteratively including results of previous predictions as features for the subsequent classifier. For the identification of dual-kinase inhibitors, predictions of two single-target SVM classifiers were combined, representing a combinatorial SVM (C-SVM) strategy [25]. Predictions were then carried out for 11 combinations of dual inhibitors for nine kinase cancer targets. Here, the C-SVM approach yielded lower false positive and comparable true positive rates for dual inhibitors compared to other ML methods [25]. C-SVM models generated on the basis of single-target inhibitors were also successfully applied to detect dual-target serotonin reuptake inhibitors [26]. Furthermore, the potential of combining single-target SVM models for multi-class predictions has also been demonstrated [27, 28]. For instance, combinations of single-target SVM models were used to predict profiling matrices of 429 compounds on 24 kinases. Here, SVM calculations were prone to false negatives but overall more predictive than other ML approaches [28]. In addition to using combinations of single-target classifiers, SVM modeling can also be adapted for predicting multi-target interactions directly. This requires the application of descriptors accounting for ligand-target pairs including, for example, representations combining compound properties with protein sequence data or structural descriptors [29]. SVM models are then trained to distinguish true ligand-target pairs from random combinations. Ligand-target interactions can also be modeled by applying different kernel functions to separately evaluate compound and target similarity and then combine these components, as illustrated in Fig. 3. For interaction predictions using such product kernels, a variety of ligand- and target-based kernel functions were developed [29, 30].

### New targets

Interaction predictions can also be attempted to identify active compounds for targets for which no ligands are known. For example, similarity searching has been applied to detect compounds active against such “orphan” targets using reference molecules from homologous targets [30]. The underlying idea is that new targets can be inferred on the basis of ligand similarity. This principle is also applicable to SVM modeling. For example, linear combinations of SVM models (LC-SVM) using compound-target kernels have been used to predict novel ligands for orphan targets [31]. Here, the performance of alternative compound-target kernels with different protein representations was often comparable and it was shown that ligand similarity and nearest neighbor relationships between known active and test compounds often determined correct SVM predictions [32].

### Activity cliffs

**Fig. 4:**
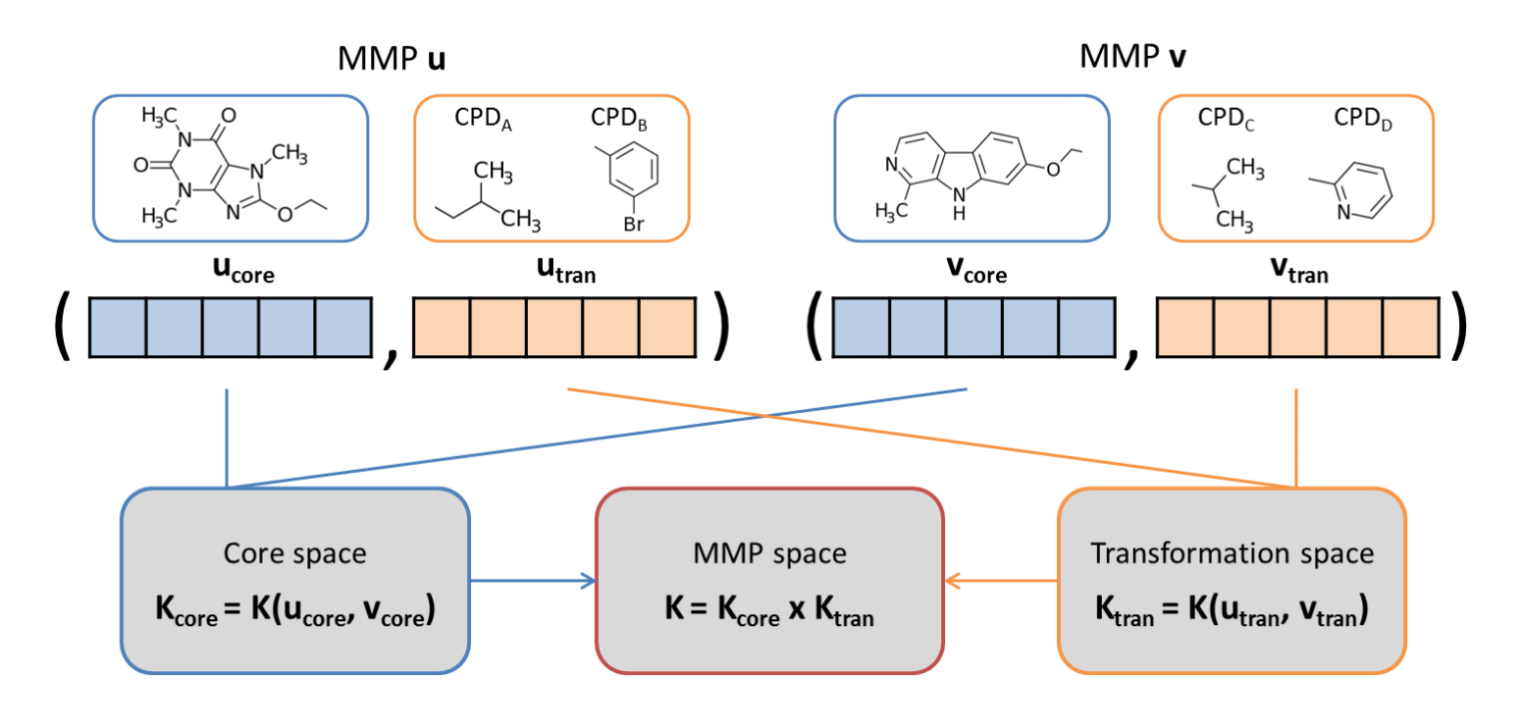
**MMP kernel.** Core (orange) and transformation (blue) descriptors are concatenated to represent an MMP and separately calculate core and transformation similarity. The common core of an MMP is represented using a fingerprint while the transformation is encoded using the concatenation of exchanged substructures or the difference between them. Then, the product of the core and transformation kernels is calculated

SVM has also been applied for the prediction of activity cliffs (ACs) consisting of pairs of structurally similar compounds with large potency differences against a given target [33, 34]. For ACs, similarity of compounds in pairs can be accounted for in different ways including, for example, the calculation of fingerprint (Tanimoto) similarity or substructure-based similarity [34]. For systematic assessment of substructure-based similarity, matched molecular pairs (MMPs) can be determined. An MMP is defined as a pair of compounds that are only distinguished by a chemical modification at a single site [35]. A modification corresponds to the exchange of a pair of substructures, termed a chemical transformation [35]. Accordingly, compounds forming an MMP share a common core and are distinguished by a given transformation. MMPs formed by compounds with large potency differences of at least 100-fold have been classified as ACs, termed MMP-cliffs [36]. For ML, prediction of ACs is principally challenging because training and test instances are compound pairs instead of individual compounds. ACs were first correctly predicted using SVM modeling, given the opportunity to design specialized kernel functions for MMPs [36], in analogy to combined compound-target kernels. The design of MMP kernels accounting for core and transformation similarity is illustrated in Fig. 4. SVM models derived using these kernel functions were applied to accurately predict MMP-cliffs for different compound activity classes by distinguishing them from corresponding MMPs capturing no significant potency differences [36]. Using such MMP kernels, potency differences between compounds forming MMP-cliffs were also successfully predicted using SVR [37]. Furthermore, as an alternative, SVM and SVR were also used to predict MMP-cliffs on the basis of condensed graphs of reaction representations [38].

## Pros and cons

In addition to standard applications of ML in chemoinformatics and drug discovery such as compound classification or property prediction, SVM has been adapted for a number of specialized applications, as discussed above, reflecting the versatility of the approach. SVM and RF have become preferred ML approaches in chemoinformatics in the pre-DL era, both for class label predictions and regression modeling, given their reliable performance in many standard applications. Compared to other ML methods, SVM has some advantages, but there are also potential caveats that must be carefully considered.

One of the strengths of SVM is the availability of a regularization hyper-parameter C that avoids overfitting, if appropriately optimized. Internal cross-validation with independent performance tests can be carried out to optimize C hyper-parameter settings. While (D)NNs contain many more hyper-parameters than SVM and RF, proper optimization of C is essential in order to generalize SVM models. RF is methodologically less complex than SVM modeling but restricted to consensus predictions. For SVM/SVR, a variety of kernel functions can be selected and modified, depending on the specific requirements of prediction tasks. For predictions relying on compound similarity, the Tanimoto kernel has become a function of choice [16], as mentioned above. Other strengths of SVM include that it solves a convex quadratic optimization problem yielding solutions approaching a global optimum [39]. In addition, the use of high-dimensional data is feasible, even in combination with small sample sizes. Furthermore, given the dependence of SVM/SVR models on SVs, not all the training data points are required for predictions. This characteristic makes SVM more memory-efficient compared to other methods that require computation of similarities or distances between all training instances.

SVM also has intrinsic limitations. Although predictions relying on SVs are generally fast, SVM learning also becomes computationally demanding when very large data sets are investigated. This is, however, rarely the case in standard chemoinformatics applications, as further discussed below. Regardless, SVM model quality is generally sensitive to the composition and size of training sets [40], which needs to be considered on a case-by-case basis. Notably, SVM is not a probabilistic approach [39], setting it apart from Bayesian modeling. For binary SVM classification, output probabilities –if desired– can be derived through logistic regression on SVM scores, which requires additional cross-validation on the training data.

**Fig. 5:**
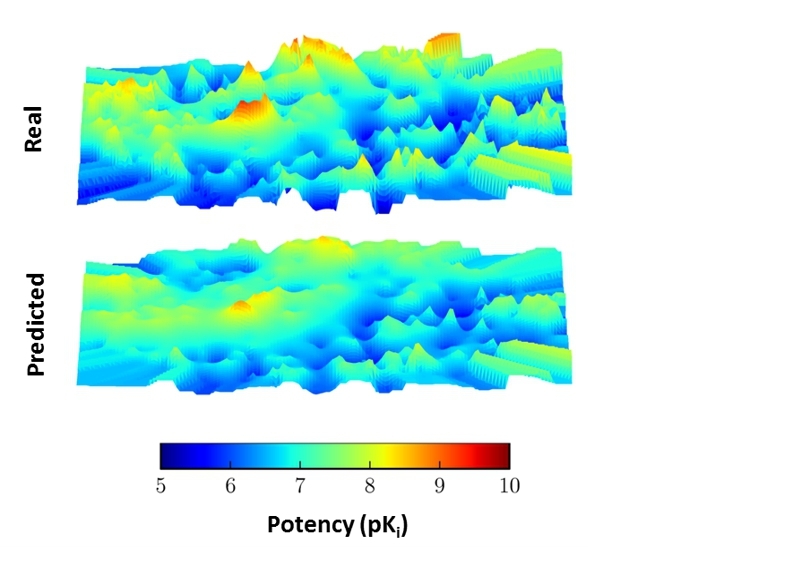
**Activity landscapes.** For a set of specifically active compounds, three-dimensional activity landscapes are generated using experimentally determined potency values (top) or potency values predicted by SVR (bottom). A color gradient accounts for the range of potency (pK_i_) values. Peaks in the “real” activity landscape represent ACs including most potent data set compounds

Limitations also apply to SVR. Importantly, SVR models often under-predict the potency of most potent data set compounds [14]. This tendency can be illustrated using three-dimensional activity landscapes of compound data sets, which are constructed on the basis of pair-wise compound distances in feature spaces mapped to an x,y-plane combined with an extrapolated potency surface added as a third dimension [41]. Figure 5 shows a representative example. SVR potency predictions “flatten” the activity landscape that is based upon experimental values by reducing the magnitude of ACs. This is a direct consequence of classifying most potent compounds as “outliers” in SVR and under-predicting their potency values. Last but not least, different from decision tree methods but similar to (D)NNs, SVM/SVR predictions have “black-box” character and are difficult to understand [42]. Accordingly, various approaches have been developed to aid in the interpretation of SVM/SVR decisions. These include the extraction of rules from models [43], identification of SVs dominating predictions [44], visualization of individual feature contributions [45], and determination of feature weights [46]. These developments complement general approaches to rationalize ML predictions [47, 48].

## Perspective

Since the early 2000s, SVM has evolved to be one of the premier ML approaches in chemoinformatics and drug discovery, together with decision tree methods and probabilistic modeling. These approaches largely replaced (shallow) NNs, which were popular early on in chemoinformatics, and have dominated ML predictions of compounds and molecular properties over the past decade. With the rise of DL in many areas of science, much attention in chemoinformatics and drug discovery is currently focused on DNNs. This also raises the question if ML approaches such as SVM or RF might be replaced by DNNs going forward. This will most likely not be the case, for several reasons. DNNs have made the strongest impact in fields were large volumes of low-resolution or unstructured data are available for modeling and where representation learning plays an important role. Often cited examples include image analysis or natural language processing. By contrast, early-phase drug discovery –dominated by chemistry and biological assays or screens– is not a data-rich field. For many standard applications such as compound classification or property prediction, confined data sets and well-defined molecular representations are available. These conditions do not play into the strengths of DL and, consequently, there is little, if any advantage of DNNs over SVM or decision tree methods in such cases. Thus, while DL using DNNs has opened the door to addressing a number of prediction tasks that were difficult to tackle using other ML approaches (such as, for example, large-scale synthesis prediction or generative molecular design), there are all reasons to anticipate that SVM will continue to be an approach of choice for many chemoinformatics applications, given its typically high performance in compound classification and property predictions on the basis of limited training data. This also applies to virtual compound screening for drug discovery. In addition, SVR will continue to be a method of choice for non-linear QSAR modeling (despite its limitations, as discussed above), especially during compound optimization where available data are usually sparse. Furthermore, as a kernel-based methodology, the adaptability and versatility of SVM for specialized applications will continue to be important for the field going forward.
